# Functional bio-packaging enhanced with nanocellulose from rice straw and cinnamon essential oil Pickering emulsion for fruit preservation

**DOI:** 10.3762/bjnano.16.91

**Published:** 2025-08-04

**Authors:** Tuyen B Ly, Duong D T Nguyen, Hieu D Nguyen, Yen T H Nguyen, Bup T A Bui, Kien A Le, Phung K Le

**Affiliations:** 1 Institute for Tropical Technology and Environmental Protection, Ho Chi Minh City, Vietnam; 2 Faculty of Chemical Engineering, Ho Chi Minh City University of Technology (HCMUT), VNU-HCM, Ho Chi Minh City, Vietnam; 3 CIRTECH Institute, HUTECH University, Ho Chi Minh City, Vietnam

**Keywords:** cinnamon essential oil, fruit preservation, Pickering emulsion, polyvinyl alcohol

## Abstract

Biopackaging materials are gaining significant attention compared to traditional synthetic polymers thanks to their biodegradable and biocompatible nature to be used in food, pharmaceutical, and cosmetic industries. The current major gaps in research regarding these biopackaging materials are their low mechanical strength and the introduction of functional additives to enhance their range of applications. In this paper, a biopackaging material is formulated using polyvinyl alcohol with glycerol as a plasticizer, rice straw-derived nanocellulose as a mechanical property enhancer and cinnamon essential oil Pickering emulsion as the main functional ingredient for strawberry preservation. With the combination of nanocellulose and Pickering emulsion, this study finds that the packaging material exhibits good heat-resistance, mechanical, and water-barrier properties. At an emulsion concentration as low as 10% (v/w) in the casting solution, high UV absorbance capacity (up to 100% UVC), high antibacterial activity (92.4% *Escherichia coli* inhibition), and good antioxidative properties (up to 43% DPPH radical scavenging) were observed. These bioactive properties and the inherent moisture barrier property of the packaging material are utilized for strawberry preservation with a significant preservation time of 21 days compared to control samples that start to grow a white fungus on day 11. This combination of biopackaging with a naturally derived functional additive is proven to be effective in preserving fruits, especially easily spoiled ones like strawberries.

## Introduction

Biopackaging materials are alternative materials to traditional oil-based plastic packaging that help mitigate environmental and health concerns relating to food preservation. They are used particularly in the food, pharmaceutical, and cosmetic sectors thanks to their good mechanical properties, high biocompatibility, and biodegradability [1–3]. Polyvinyl alcohol (PVA) has been shown to provide better biodegradability compared to other polymers such as polyethylene, polyvinyl chloride, and polystyrene [4–6] due to its secondary alcohol groups being susceptible to enzymatic oxidation and its water solubility enhancing microbial access [7]. PVA also exhibits superior biocompatibility as evidenced by its high safety threshold (LD_50_ of 15–20 g·kg^−1^), low systemic absorption, absence of mutagenic effects, and established medical applications, making it a reliable choice for biopackaging without concerns of harmful degradation byproducts [8–9]. One problem is that the hydrophilic structure of PVA gives it a high water-solubility, water uptake, and worse mechanical properties [10–11]. Different filler and nanomaterials including silica [12–14], graphene [15–16], and metals [17–18] have been added to PVA to develop composite materials with superior properties.

Compared to other inorganic nanomaterials, nanocellulose (NC) has been noted to be a highly potential sustainable and bio-based filler that can be obtained from otherwise wasted agricultural byproducts like rice straw [11,19]. NC can enhance polymer matrix properties, including tensile strength, elasticity, and thermal stability, due to its high surface area, hydroxy groups forming hydrogen bonds, and excellent dispersion within the matrix [20–21]. This improvement is attributed to interaction between hydroxy groups in NC and PVA, effectively reinforcing the nanofiber structure and providing better resistance to moisture-induced degradation [22]. Studies have shown that the addition of NC can significantly enhance the mechanical properties of PVA where tensile strength has been reported to increase from around 20 MPa to well over 30 MPa [23–24]. Similarly, the water vapor permeability has also been reported to change. It generally decreases with the incorporation of nanocellulose [24], thereby preventing the drying out of the fruits to be preserved, which can greatly reduce their quality [25].

One factor to be considered with this reduced permeability is that the trapped moisture with the inherent highly nutritious components of the fruits can also inversely introduce the growth of microbials, thereby facilitating the spoiling process [26]. This pushes recent research to focus on the incorporation of bioactive ingredients to introduce antimicrobial and antioxidation properties that can offer extended shelf life [27–29]. Essential oils are great candidates and possess a variety of bioactive properties while maintaining a high biocompatibility [30]. Cinnamon essential oil (CEO) is one of the best-known essential oils with strong activity against a range of Gram positive and Gram negative bacteria thanks to the cinnamic aldehyde and eugenol content in its composition [31–33]. The effectiveness of incorporating CEO in the preservation of fruits has been demonstrated. The preservation of mangoes was extended by 7 days [34] and the color of persimmons was maintained up to 63 days in cold storage [35].

The major challenge of using CEO is its hydrophobic nature, which prevents it from blending with the biopackaging casting solution, and its volatile and unstable structure, which can lead to significant loss of efficacy over time [36]. Encapsulation strategies, like Pickering emulsions (PEs) stabilized by bio-based particles, address these challenges [37]. Nanocellulose is a promising candidate for stabilizing Pickering emulsions because of its high surface area, nanoscale dimensions, and amphiphilic nature [20]. This not only improves the dispersibility of essential oils in biopolymer matrices but also enhances the overall compatibility and performance of the biopackaging material [22]. Our previous research has demonstrated that nanocellulose can effectively encapsulate CEO into a PE with small particle sizes (<700 nm), high stability, and strong antimicrobial and enhanced DPPH inhibition properties [38].

This study represents the first attempt to integrate rice straw-derived nanocellulose both as a biopackaging reinforcing filler and as a carrier for cinnamon essential oil Pickering emulsions (PE-CEO) into a single biopackaging material. While earlier studies have focused on either nanocellulose as a reinforcement or Pickering emulsions for bioactive delivery, their combined potential in scalable solutions has not been explored. By integrating nanocellulose and essential oils, biopackaging can achieve superior mechanical strength, enhanced bioactivity, and greater environmental sustainability. This synergy paves the way for scalable, eco-friendly solutions that align with industrial demands for highly functional packaging materials. To validate its real-world applicability, the biopackaging was tested on strawberry, a nutritious fruit that is highly susceptible to microbial contamination and mold growth [39] to highlight its potential to extend shelf life and improve postharvest quality. The findings indicate that this bioactive film could serve as an effective alternative to conventional packaging, offering both sustainability and enhanced food safety [30].

## Results and Discussion

### Physicochemical properties

**Figure 1:**
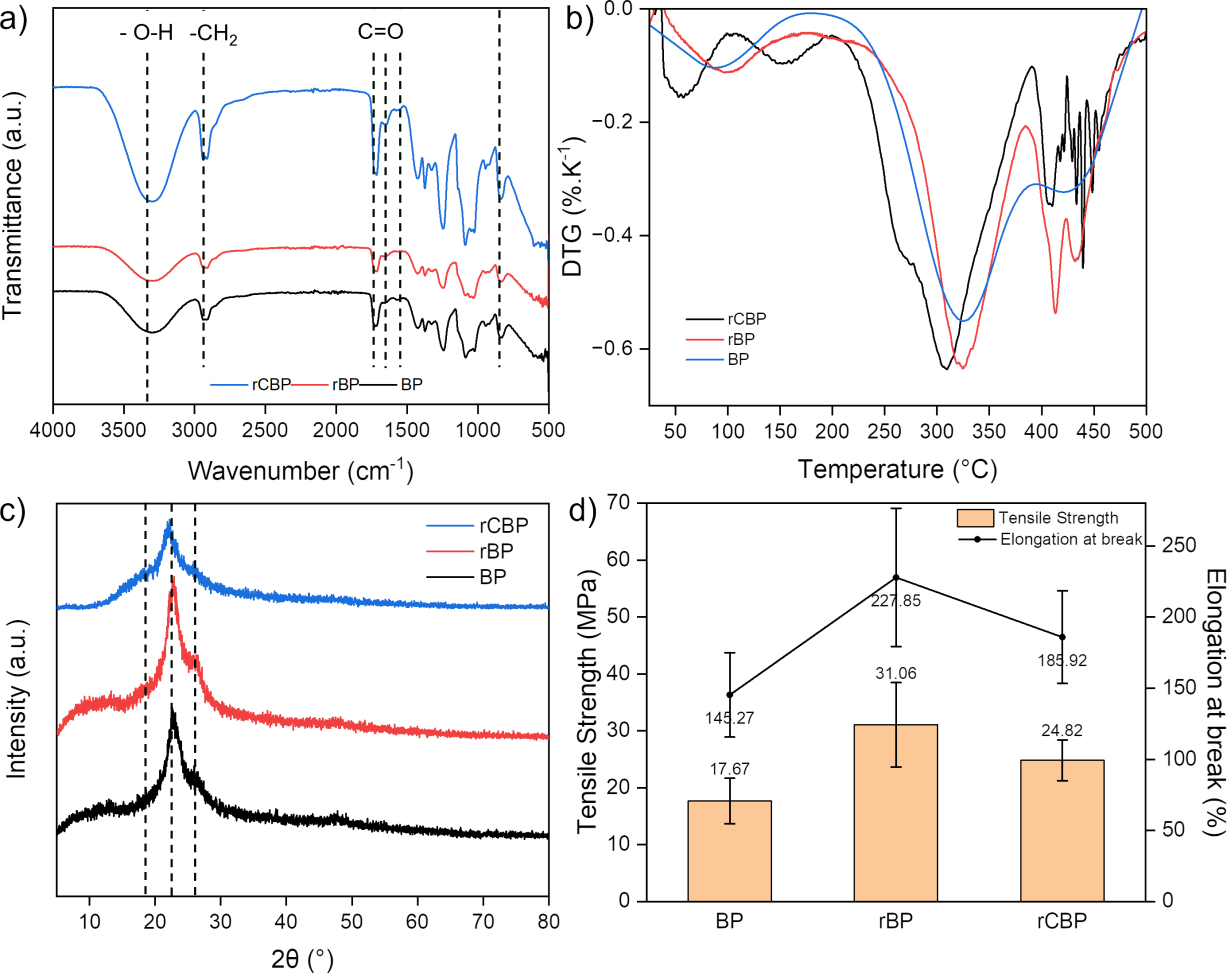
Effect of NC and PE-CEO on properties of biopackaging materials. (a) FTIR spectra, (b) DTG, (c) XRD, and (d) tensile strength and elongation at break.

FTIR spectroscopy (Figure 1a) was used to study the PVA/glycerol biopackaging (BP), NC-reinforced BP (rBP), and PE-CEO-containing rBP (rCBP) composite films. BP exhibited characteristic peaks at 3330 cm^−1^ (O–H stretching), 2900 cm^−1^ (C–H stretching), and 1420 cm^−1^ (C–H bending), which align with the chemical structure of PVA [22,24]. When NC was added, the O–H stretching peak became broader and shifted slightly to 3315 cm^−1^, which can be attributed to the hydrogen bonding between PVA and NC [24]. For rCBP, additional peaks appeared at 1510 and 1745 cm^−1^, corresponding, respectively, to the aromatic rings and C=O stretching of cinnamaldehyde of CEO [31]. The little difference found between rCBP and rBP suggests a low effect of CEO on the biopackaging, which can be explained either by the low concentration of CEO or the trapping effect of the polymer matrix on CEO [31].

XRD analysis (Figure 1c) shows that all biopackaging samples exhibit only large peaks at around 22° and 26°, which correspond to the (101) and (200) planes of the PVA structure [40]. It is to note that a slight peak shift to higher angles can be seen for rBP compared to BP, which indicates a decrease in interplanar spacing, suggesting that NC has infiltrated the PVA lattice structure. For rCBP, with the addition of PE-CEO that has a larger particle size (≈700 nm [38]), the peaks were shifted towards lower angles and the peak intensity was visibly lower. This may be related to the disruption of the crystalline structure of PVA after encapsulation of essential oil. For rBP and rCBP, which also contains NC, a small rise in the XRD signal can be seen at around 18°, which may be from the (110) plane of the Iβ cellulose crystalline phase [32].

Differential thermogravimetry (DTG) results (Figure 1b) revealed three distinct stages of weight loss. The first stage (30–130 °C) was attributed to water evaporation, consistent with the hydrophilic nature of PVA [41]. The second stage (260–380 °C) involved polymer chain degradation and volatile compound release [42]. At temperatures above 380 °C, carbonaceous residues decomposed. The addition of NC shifted the degradation onset temperature to a higher range, indicating improved thermal stability. This could be due to the rigid nature of NC particles, which enhance the structural integrity of the composite, delaying its decomposition [24]. CEO did not significantly alter the thermal degradation pattern, confirming that its incorporation did not compromise the thermal stability of the samples. These findings indicate that PVA-NC films are thermally suitable for applications like food packaging, where moderate temperature resistance is required.

Figure 1c illustrates the mechanical properties of biopackaging films, including tensile strength and elongation at break. The incorporation of NC significantly enhances the tensile strength of the films by more than 40%, reaching 31.06 MPa, due to the reinforcing effect of nanocellulose within the polymer matrix [41]. The presence of PE-CEO slightly decreases tensile strength and elongation at break to 24.82 MPa and 190%, respectively [43].

**Figure 2:**
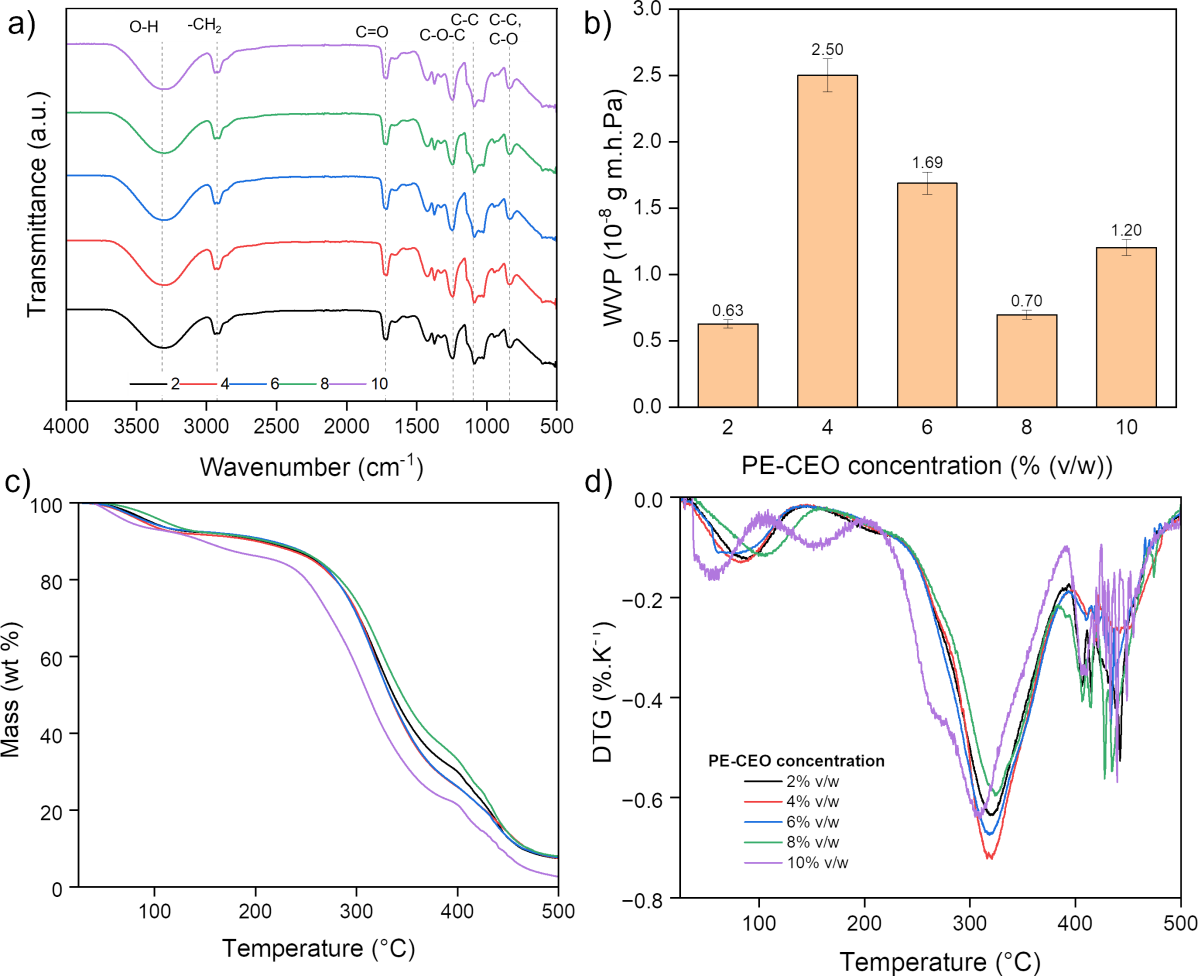
Effect of cinnamon essential oil Pickering emulsion concentration (2%, 4%, 6%, 8%, and 10% (v/w)) on properties of biopackaging materials. (a) FTIR spectra, (b) water vapor permeability, (c) TGA, and (d) DTG.

FTIR spectra of the biopackaging films containing different concentrations of CEO are shown in Figure 2a. The characteristic peaks confirm the presence of functional groups associated with PE-CEO and polymer interactions. The broad absorption band around 3300 cm^−1^ corresponds to O–H stretching vibrations, indicating hydrogen bonding between the biopolymer and PE-CEO components [24]. The intensity of the peaks at 1730 cm^−1^ (C=O stretching from ester or carboxyl groups) and 1600 cm^−1^ (C=C stretching of aromatic rings) changes with increasing PE-CEO concentration, suggesting interactions between PE-CEO and the polymer network [44]. These variations indicate successful incorporation of PE-CEO, which could influence the physicochemical properties of the films.

Thermogravimetric analysis (TGA) (Figure 2c) shows how increasing the PE-CEO concentration may affect the thermal stability of the sample. At lower PE-CEO concentration, almost no variation between the rCBP samples can be observed. At 10% (v/w) PE-CEO concentration, a notable decrease in mass can be observed, which is attributed to the high volatility of CEO and the fact that the high oil concentration in the biopackaging matrix makes it harder to fully load and encapsulate CEO. All biopackaging samples maintain masses of more than 50 wt % at up to 300 °C, indicating that these biopackaging are thermally suitable for applications like food packaging where moderate temperature resistance is required.

The effect of CEO concentration on water vapor permeability (WVP) of the biopackaging films is illustrated in Figure 2b. WVP is a critical factor for packaging applications, influencing barrier properties against moisture transmission. The results show that adding CEO reduces WVP values compared to the control. At 4% (v/w) PE-CEO, WVP decreases by approximately 23.5%, reaching 4.12 × 10^−12^ g·m^−1^·s^−1^·Pa^−1^ due to increased hydrophobicity and reduced polymer chain mobility [43]. When CEO content exceeds 8%, WVP slightly increases to 5.27 × 10^−12^ g·m^−1^·s^−1^·Pa^−1^, likely due to structural heterogeneity and phase separation within the polymer matrix [45]. These findings suggest that an optimal PE-CEO concentration exists to balance water resistance and mechanical stability, making PE-CEO-infused films promising candidates for biopackaging applications requiring controlled moisture permeability.

**Figure 3:**
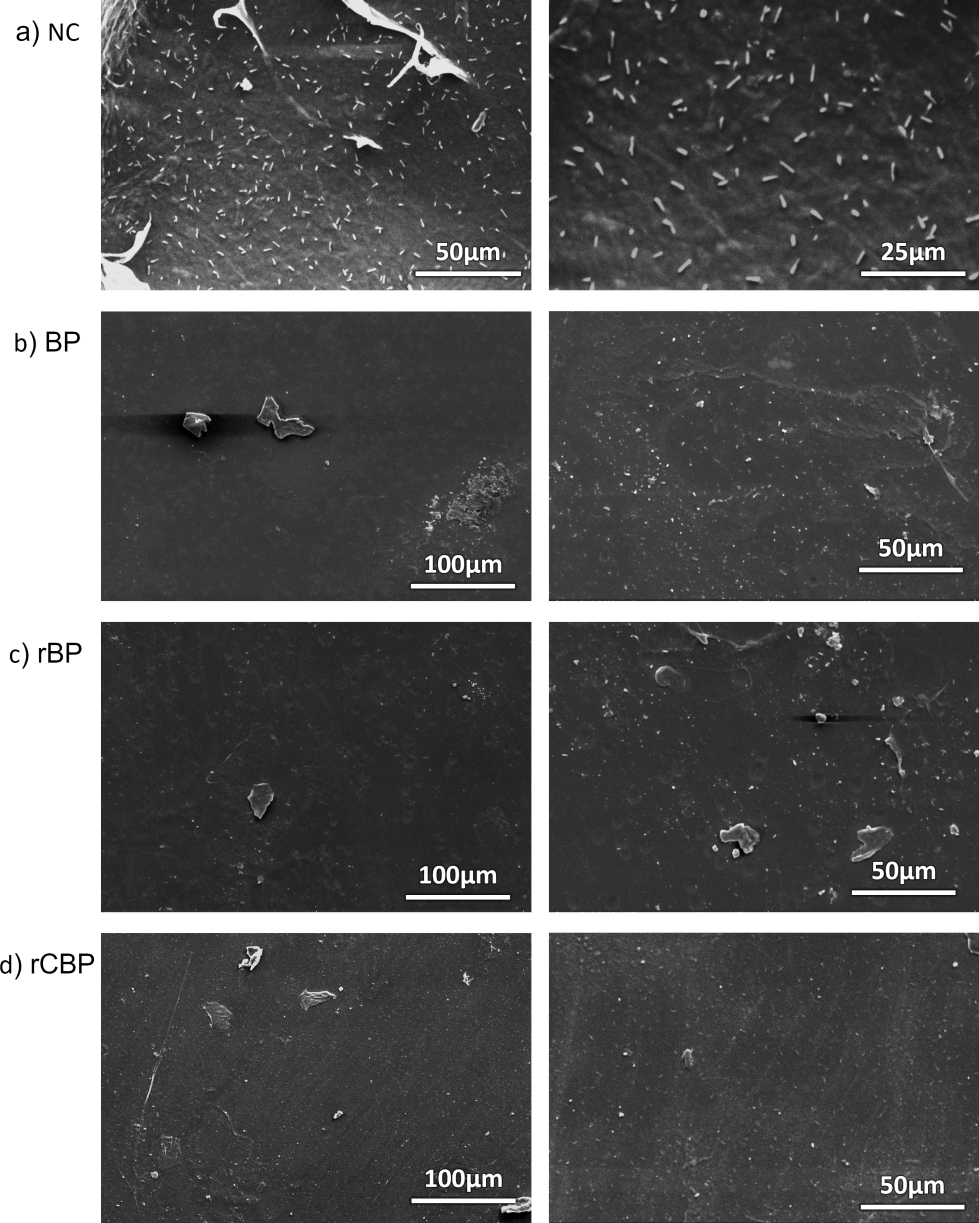
SEM images of the samples. (a) Nanocellulose (NC), (b) biopackaging (BP), (c) NC-reinforced biopackaging (rBP), and (d) reinforced biopackaging with PE-CEO at 6% (v/w) (rCBP).

The surface morphology of the biopackaging materials was examined using scanning electron microscopy (SEM) (Figure 3). As shown in our previous research, nanocellulose in suspension has a size of around 20–30 nm and a length below 300 nm, and size increase due to structural collapse would be seen upon drying [24,46]. A SEM image of the nanocellulose sample is given in Figure 3a. After freeze-drying, some coagulation occurred, increasing the size of the sample to reach ≈4 µm in diameter and ≈1 μm in length.

Pure BP films (Figure 3b) exhibited a relatively smooth surface with visible small pores, indicative of weak intermolecular interactions. The incorporation of NC into the PVA matrix significantly reduced surface roughness and void formation, suggesting enhanced structural integrity due to the strong hydrogen bonding between NC and PVA (Figure 3c) [22]. The addition of CEO further modified the film structure, forming a more compact and homogenous surface, which likely contributed to improved mechanical properties and moisture resistance (Figure 3d) [31].

### Bioactive properties

**Figure 4:**
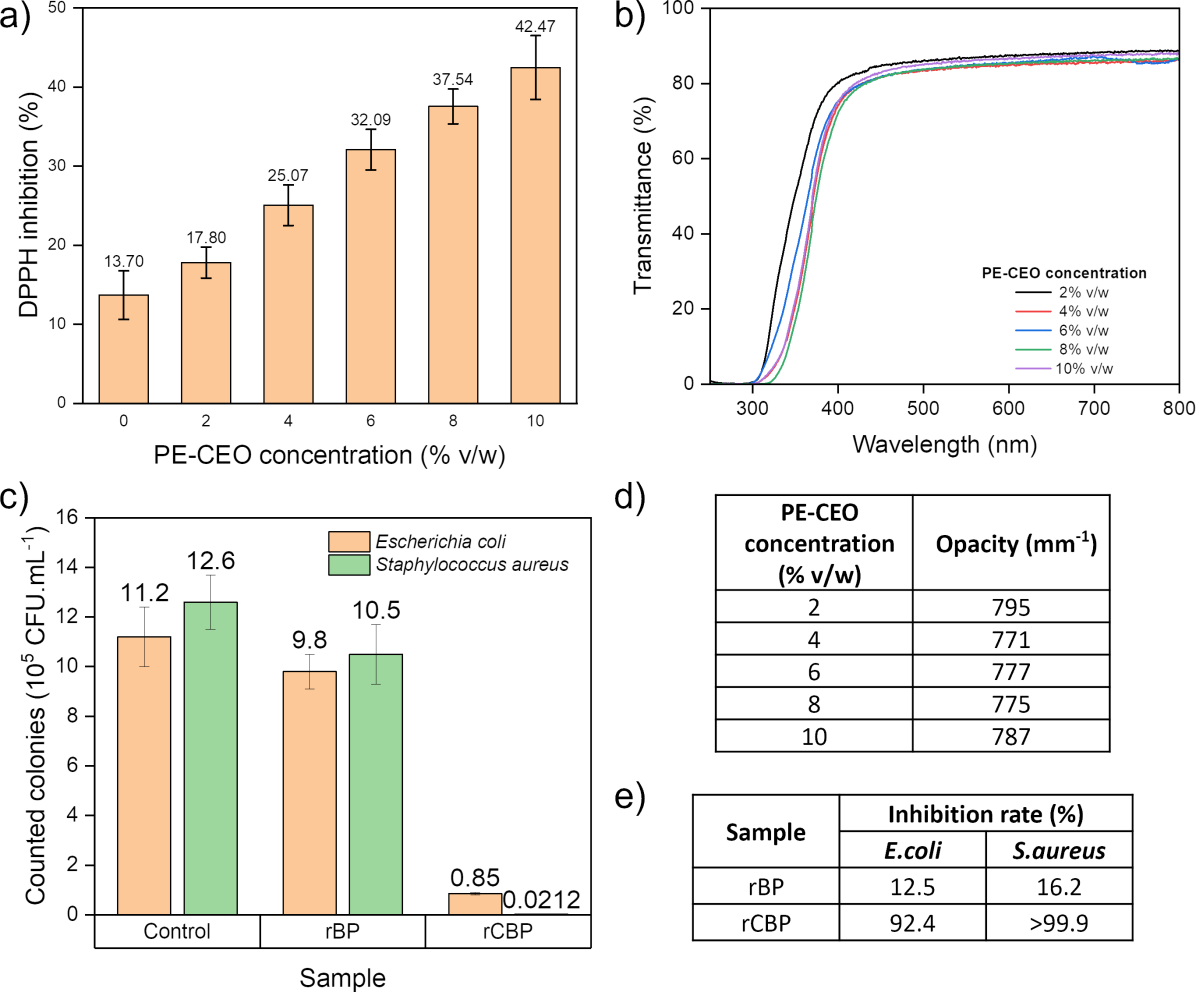
Bioactive properties of the sample. (a) DPPH scavenging activity, (b) UV–vis transmittance, and (d) opacity at different PE-CEO concentrations (2%, 4%, 6%, 8%, and 10%(v/w)). (c) Counted colonies and (e) inhibition rate from time-kill analysis using rCBP samples at 10% (v/w) PE-CEO after 24 h against *E. coli* and *S. aureus*.

The DPPH radical scavenging (Figure 4a) showed a linear increase in scavenging activity with increasing CEO concentration, reaching a maximum of 43% at 10% (v/w) CEO incorporation. This trend suggests a direct correlation between the phenolic content of CEO and its ability to inhibit DPPH free radicals [47]. The antioxidative performance of the films aligns with previous studies on essential oil-infused biopolymer matrices, confirming their potential to enhance food packaging stability by preventing oxidative degradation [48].

With increasing CEO concentration, transmittance decreased significantly in the UV region (300–400 nm) (Figure 4b), demonstrating strong UV blocking capability [49]. The transmittance even dropped to near zero in the UVC range (280–315 nm). Above 400 nm, the transmittance was higher but remained below 100%, highlighting the dual function of PE-CEO in absorbing harmful UV rays while partially limiting visible light transmission (Figure 4d). This makes rCBP ideal for preserving light-sensitive products such as fruits and vegetables, protecting them from photodegradation and extending their shelf life [24]. This effect can be attributed to the presence of cinnamaldehyde and other phenolic compounds in CEO, which act as natural UV absorbers [50].

The antimicrobial properties of the biopackaging were assessed by a time-kill assay against *E. coli* and *S. aureus* after 24 h of exposure (Figure 4c,e). rBP exhibited slight inhibitions of *E. coli* and *S. aureus* (<20%): This can be due to the presence of hydroxy groups in PVA, which can disrupt hydrogen bonds and dissolve the peptidoglycan membranes of the bacteria [51]. CBP showed a drastic time-kill effect of more than 90% for both types of bacteria, attributed to the potent antibacterial functional groups in CEO (cinnamaldehyde and eugenol) [33,52].

**Figure 5:**
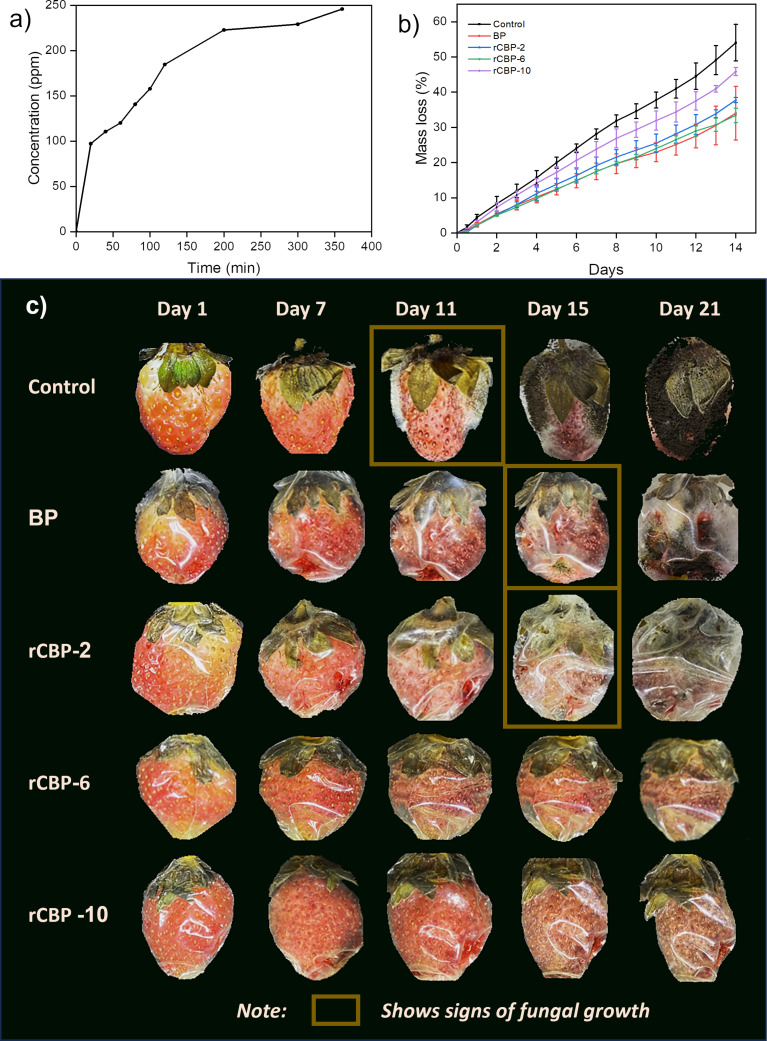
Results on strawberry preservation. (a) Release profile of CEO from rCBP-10, (b) mass loss record by days, and (c) appearance of the fruits after 1, 7, 11, 15, and 21 days of preservation for control (commercial PVC), BP, and reinforced biopackaging with PE-CEO (rCBP) at different concentrations (2%, 6%, and 10% (v/w)).

The release of CEO from the films was studied over time (Figure 5a). The release pattern had two phases, namely, a fast initial release of 42% within the first 100 min, followed by a slower phase up to 300 min. The fast release happened because some CEO was near the surface of the film, while the slower phase was controlled by how CEO moved through the polymer.

Mass loss and appearance of the strawberry samples when covered by the biopackaging material are presented in Figure 5b,c. Strawberry is in the focus of this research since its moist and soft structure can support the growth of microorganisms, making them spoil as early as 1–2 days after harvest without any processing [39]. Several attempts have been made that could extend strawberry preservation only up to 6, 9, and 10 days, respectively, with PVA/chitosan/1,8-cineole/cyclodextrin [53], PVA with lids [54], and modified atmosphere polypropylene [55]. In this research, without any biopackaging, the strawberry would grow moldy right on day 11 and become dryer and reduced in size, which led to the fastest decrease in mass. In contrast, at no or very low concentration of CEO (up to 2% (v/w)), the time until fungal growth was extended to day 15, which is due to the bioactive properties of CEO. When increasing the CEO concentration to 6% and 10% (v/w), no fungal growth was recorded up to day 21. It is important to note that at low CEO concentrations, the biopackaging acts as a moisture barrier to prevent moisture escape from the sample, making the sample wet and foggy at day 21. At higher CEO concentrations, the biopackaging enables moisture escape to further prevent the proliferation of fungi and contaminations. This led to slightly higher mass loss upon preservation in these samples. All in all, these results showed that rCBP biopackaging materials are effective in extending the strawberry preservation time up to 21 days.

## Conclusion

This study successfully developed an innovative biopackaging material by integrating rice straw-derived NC and cinnamon essential oil stabilized within a Pickering emulsion (PE-CEO) into a polyvinyl alcohol matrix. The incorporation of NC has been shown to enhance thermal stability, mechanical strength, and water vapor barrier property of the films, while the addition of PE-CEO imparted strong UV blocking, antimicrobial, and antioxidative properties. TGA and mechanical strength tests confirmed that NC improves the structural integrity. The controlled release of CEO helped to ensure prolonged bioactive effects, providing a dual-function material suitable for food preservation applications. The resulting films demonstrated their practical potential by extending the shelf life of strawberries to 21 days, compared to 11 days for unwrapped samples, therefore highlighting the potential of combining renewable materials and natural functional additives to create scalable, eco-friendly packaging solutions.

## Experimental

### Materials

Rice straw used for NC synthesis was provided by Loc Troi Group from An Giang province, Viet Nam. Cinnamon essential oil (CEO) was obtained from pure *Cinnamomum verum* bark through steam distillation by Notessen Co. Ltd. (Viet Nam) and exhibited a cinnamaldehyde content greater than 96%. All chemicals, including sulfuric acid (H_2_SO_4_), polyvinyl alcohol (PVA), and glycerin (C_3_H_8_O_3_), were obtained from commercial sources and used as received.

### Material synthesis

#### Nanocellulose synthesis process

Rice straw served as the source material for nanocellulose production. Following a previously established procedure [46], two alkaline pretreatment steps and a bleaching stage were used to extract cellulose from the straw. In the first acid hydrolysis step, the cellulose was treated with 62% H_2_SO_4_ solution at a solid-to-liquid ratio of 1:12 (g·mL^−1^), continuously stirred at a temperature of 40–42 °C for 2 h. The reaction was then quenched by 10-fold dilution of the solution. The solution was washed by centrifugation three times and filtered through a 10 μm nylon mesh filter membrane. In the neutralization step, the solution was neutralized by dialysis with deionized water, which was replaced every 6 h, and sonicated using a Hielscher UP400St ultrasonic homogenizer (Germany) to create the NC suspension. Our previous research has stated that the size of the NC crystals was around 20–30 nm in width and 300 nm in length [24,46]. The NC concentration in the suspension was analyzed by dripping 3 mL of the suspension onto a pre-dried Petri dish. Subsequent drying was performed in a convection oven at 60 °C until a constant weight was achieved. The NC content was then determined by applying Equation 1:


[1] $$\%m_{\text{NC}}=\left(m_{2}-m_{0}\right)/\left(m_{1}-m_{0}\right),$$

where *m*_0_ represents the mass of the dried petri dish, *m*_1_ is the mass recorded after adding the suspension, and *m*_2_ is the mass measured after the sample had dried.

#### Stabilization of oil-in-water Pickering emulsions using nanocellulose

The cinnamon essential oil Pickering emulsions (PE-CEO) were prepared by combining essential oil at 15% (v/v) with nanocellulose suspensions at 0.6% (w/v). The mixture of essential oil and nanocellulose suspensions was then sonicated using a Hielscher UP400St ultrasonic homogenizer (Germany). The sonication process was conducted at a power of 200 W with each cycle consisting of 2 min of sonication followed by 2 min of rest for a total of six cycles, corresponding to a total sonication time of 12 min.

#### Preparation of essential oil Pickering emulsion containing biopackaging

PVA was dispersed in water at 6% (w/v) using a magnetic stirrer with heating at 80 °C for 3 h and 1 mL of glycerol was added to create the biopackaging (BP) film-forming solution [24]. NC suspension at a *m*_NC_/*m*_PVA_ ratio of 6% (w/w) was added to form the NC-reinforced BP solution (rBP). PE-CEO was added at *m*_PE_/*m*_PVA_ ratios of 2%, 4%, 6%, 8%, and 10% (v/w) for the corresponding NC-reinforced PE containing biopackaging (rCPB-2, rCPB-4, rCPB-6, rCPB-8 and rCPB-10). The film solution was then sonicated in a water bath to remove any bubbles and cast onto a mold and dried at 70 °C for 12 h. All concentrations were based on the mass of PVA.

### Characterization

#### Physicochemical properties

Fourier transform infrared spectroscopy (FTIR) spectra were recorded in the range of 4000–500 cm^−1^ using a Bruker ALPHA II spectrometer (Germany) at a spectral resolution of 4 cm^−1^.

Thermal stability was determined using thermogravimetric analysis (TGA) and differential thermogravimetric analysis (DTG). Samples were heated from 25 to 500 °C at a rate of 10 K·min^−1^ under N_2_ atmosphere (50 mL·min^−1^) in a METTLER TOLEDO 3+ Large furnace (Switzerland).

X-ray diffraction (XRD) was performed using an Aeris Minerals Edition from PANalytical (UK) with Co radiation at 40 kV. Biopackaging samples were clipped on a 16 mm holder and the measurement was performed using a 1/8° diffraction slit.

Mechanical strength was determined at room temperature using a Testometric X350 testing machine (UK) following the ASTM D882 standard. Testing was performed at a crosshead speed of 50 mm·min^−1^ using a 1 N load cell on 1 cm × 7 cm specimens at room temperature.

Water vapor permeability (WVP) was measured using a modified ASTM E96/E96M-16 method. 90 mm diameter biopackaging samples were wrapped around cups containing 40 mL of distilled water and sealed with plastic lids with a 50 mm diameter opening. The weight of each cup was recorded every hour for 8 h to calculate the water vapor transmission rate (WVTR) and repeated three times. The water vapor transmission rate (WVTR) was calculated based on the weight loss over time (Equation 2), and WVP was subsequently determined using (Equation 3):


[2] WVTR=G/(t⋅A),

where WVTR is the water vapor transmission rate (g·h^−1^·m^−2^), *G* is the change in mass (g), *t* is the test duration (h), and *A* is the test area (m^2^).


[3] $$\text{WVP}=\left(\text{WPTR}\cdot L\right)/\Delta P=\left(\text{WVTR}\cdot L\right)/\left(S\left(R_{1}-R_{2}\right)\right),$$

where WVP is the water vapor permeability of the sample (g·m^−1^·h^−1^·Pa^−1^), *L* is the sample thickness (m), Δ*P* is the vapor pressure difference (Pa), *S* is the saturation vapor pressure at the test temperature (Pa), *R*_1_ is the relative humidity inside the dish, and *R*_2_ is the relative humidity at the cup.

The surface morphology of the biopackaging materials (BP, rBP, and rCBP) was observed using a scanning electron microscope (SEM), model Primas E (US). The samples were coated with Pt for 30 s prior to measurement.

#### Bioactive properties

**UV absorption.** Transparency and UV absorption of the films were determined using UV–vis spectroscopy in the wavelength range of 250–700 nm on a 754 STECH INTERNATIONAL spectrophotometer (China). Five samples (1 × 4 cm^2^) were measured with transparency and UV protection assessed by measuring transmittance at 600 nm and 280 nm, respectively. The opacity of the biopackaging is calculated based on its absorbance at 600 nm and its thickness as (Equation 4).


[4] $$\text{opacity}=\frac{{\text{Ab}}_{600}}{t},$$

where Ab_600_ represents the absorbance at 600 nm and *t* is the biopackaging thickness [49].

**Antimicrobial properties.** The antimicrobial activity of the films was evaluated using a time-kill method. *Escherichia coli* ATCC 25922 and *Staphylococcus aureus* ATCC 29213 were grown in tryptic soy broth and standardized to a concentration of approximately 1.5 × 10^8^ CFU·mL^−1^ (McFarland 0.5). Prior to analysis, the test films (BP, rBP, and rCBP) were sterilized under UV light for 5 min using a 30 W UVC lamp at a distance of 15 cm. Semicircle biopackaging samples (roughly 55 cm^2^, from half a Petri dish) were added to the microbial suspensions in saline water and incubated for 24 h. A control tube without the film was prepared in parallel. After 24 h of incubation at 37 °C, serial dilutions were prepared and plated on Mueller–Hinton Agar (MHA) and colony-forming units (CFU) were counted to determine the antimicrobial activity of the films. Inhibition rates were calculated according to


[5] $$I\left(\%\right)=\frac{{\text{CC}}_{\text{control}}-{\text{CC}}_{\text{sample}}}{{\text{CC}}_{\text{control}}},$$

where CC_control_ is the cell count of the control tube substance and CC_sample_ is the cell count of the sample after 24 h.

**Antioxidation properties.** The antioxidant capacity of the samples was assessed using the 1,1-diphenyl-2-picrylhydrazyl (DPPH) radical scavenging assay. Exactly 1.00 g of biopackaging samples were added into 1.8 mL of 80% methanol. Afterwards, 3.2 mL of 0.1 mM DPPH solution was added to ensure that the absorbance of the control was above 0.6, and the samples were kept in the dark for 30 min. Measurements was performed using a 754 STECH INTERNATIONAL (China) UV–vis spectrophotometer at 517 nm. The DPPH radicals scavenging ability was calculated using (Equation 6):


[6] $$I\left(\%\right)=\frac{A_{\text{control}}-\left(A_{\text{sample}}-A_{\text{color}}\right)}{A_{\text{control}}},$$

where *A*_control_ is the absorbance of the sample without the test substance, *A*_sample_ is the absorbance of the sample containing both the test substance and DPPH, and *A*_color_ is the absorbance of the sample containing the test substance without DPPH.

**Controlled release profile.** The controlled release of CEO from the films was evaluated every 20 min over 380 min by soaking exactly 1 g of film sample into 10% (v/v) ethanol solutions. The released CEO was measured by analyzing the CEO concentration in the solution through the absorbance at 290 nm using a 754 STECH INTERNATIONAL spectrophotometer following a previously established CEO standard curve [38].

#### Strawberry preservation

Strawberries were chosen for this study. Unripe strawberries, slightly green in color, were selected and soaked in 80% ethanol to eliminate potential microbial contamination on the outer surface before storage. The strawberries were wrapped in a layer of biopackaging and stored under the same refrigeration conditions at 10 °C. The weight of each strawberry, including its packaging, was recorded on the first day of the experiment. Daily photographs and weight measurements were taken to monitor changes in mass and appearance. The experiment was carried out for a period of 21 days, which is the final time where all strawberries were covered with a white layer of fungi.

## Data Availability

All data that supports the findings of this study is available in the published article and/or the supporting information of this article.
